# A Prognostic Riskscore Model Related to *Helicobacter pylori* Infection in Stomach Adenocarcinoma

**DOI:** 10.1155/ijog/5554610

**Published:** 2025-01-21

**Authors:** Jing Peng, Qi Yan, Wennan Pei, Yi Jiang, Li Zhou, Ruoqing Li

**Affiliations:** ^1^Department of General Medicine, Chongqing University Central Hospital, Chongqing Emergency Medical Center, Chongqing Key Laboratory of Emergency Medicine, Chongqing, China; ^2^Department of Gastroenterology and Hepatology, Chongqing Emergency Medical Center, Chongqing University Central Hospital, Chongqing, China

**Keywords:** *Helicobacter pylori*, immune microenvironment, prognosis, riskscore model, stomach adenocarcinoma

## Abstract

**Background:** *Helicobacter pylori* (*HP*) is associated with the development of various stomach diseases, one of the major risk factors for stomach adenocarcinoma (STAD).

**Methods:** The *HP* infection score between tumor and normal groups was compared by single-sample gene set enrichment analysis (ssGSEA). The key modules related to *HP* infection were identified by weighted gene coexpression network analysis (WGCNA), and functional enrichment analysis was conducted on these module genes. Further, the limma package was used to screen the differentially expressed genes (DEGs) between *HP*-positive and *HP*-negative STAD. The prognostic genes were obtained to construct the riskscore model, and the performance of the model was validated. The correlation between riskscore and tumor immune microenvironment (TIME) was analyzed by Spearman's method. The single-cell atlas of *HP*-positive STAD was delineated. The mRNA expression levels of the prognostic genes were verified using STAD cells, and the migration and invasion capacities of STAD cells were evaluated by using the wound healing assay and transwell assay.

**Results:** The *HP* infection score in the tumor group was significantly higher than that in the normal group. The purple and royal blue modules showed higher correlation with *HP* infection in STAD, and these module genes were enriched in the immune-related pathway. Further, five prognostic genes (*CTLA4*, *CPVL*, *EMB*, *CXCR4*, and *FAM241A*) were screened from the *HP* infection–related DEGs, which were utilized for establishing the riskscore model, with good robustness. Riskscore exhibited strong correlation with TIME in STAD. Single-cell atlas of *HP*-positive STAD revealed that *CXCR4* is highly expressed in Epithelial Cell 1, Epithelial Cell 2, and parietal cells of the tumor group. *CPVL*, *EMB*, *CTLA4*, *FAM241A*, and *CXCR4* showed high expression in STAD cells, and the silencing of *CPVL* could suppress the migration and invasion of STAD cells.

**Conclusion:** This study established a riskscore model based on *HP* infection–related genes, which could provide reference for prognostic prediction and treatment targets of STAD.

## 1. Introduction

Gastric cancer (GC) is a prevalent malignancy that originates from the epithelial cells of the stomach [1, 2]. By 2020, the incidence rate of GC ranks fifth in the world, with about 1.1 million new cases, and the mortality ranks fourth, with approximately 800,000 deaths [3]. Stomach adenocarcinoma (STAD) is the most common pathological subtype of GC, accounting for 95% [4]. STAD possesses typical heterogeneity and strong invasiveness, which usually leads to different clinical behaviors and poor outcomes [5, 6]. Numerous risk factors can influence the occurrence and development of STAD, comprising host genetics and environment, such as age, sex, *Helicobacter pylori* (*HP*) infection [7], high nitrite diet, drinking, and smoking [8]. In recent decades, great advancement has been made in the endoscopic examination, surgical treatment, and chemotherapy for early-stage STAD [9, 10]. However, because of the limited clinical symptoms and absence of routine gastroscopic examination, shatteringly, 80%–90% of STAD patients have already developed distant metastasis and progressed to an advanced stage when diagnosed [11–14]. As a consequence, the prognosis of STAD remains disappointing, and the 5-year survival rate is not exceeding 30% [15]. The lack of early detection is the main reason that affects the survival rate of STAD; thus, seeking effective diagnostic biomarkers and potential therapeutic targets is very crucial for improving the diagnosis, prevention, and treatment of STAD [16].

Microbiota plays a critical role in the human gastrointestinal tract, and ecological disorders will occur when the balance between microbiota and host is disrupted [17]. The intestinal bacterial community is reported to be closely associated with the occurrence and development of STAD [18]. Particularly, *HP* infection is regarded as the principal cause of STAD worldwide [19]. *HP* is a Gram-negative bacterium that can secrete various virulence factors, enabling it to colonize and reproduce in the extremely acidic gastric mucosa of humans [20]. In 1994, *HP* has been classified as a Class I carcinogen by the World Health Organization (WHO) [21]. The continuous *HP* infection can lead to many gastrointestinal diseases, including chronic gastritis, gastric ulcers, duodenal ulcers, polyps, and STAD [22, 23]. Due to the increasing cases of drug-resistant *HP* infections, the supervision of STAD becomes more and more difficult [24]. Eliminating *HP* can availably decrease the hazard of gastric precancerous lesions [22]. A previous study of Guo manifested that in *HP*-positive GC patients, the Shannon and richness indexes of 18 gastric microbial genera were markedly enhanced after *HP* eradication, such as probiotic *Bifidobacterium* [25]. In gastrointestinal cancers, the microbiota has been considered to be correlated with the curative effect of radiotherapy, chemotherapy, and immunotherapy, which indicated that the gastrointestinal microbiota may serve as potential targets for the treatment of STAD [26]. Therefore, understanding the relationship between *HP* infection and STAD prognosis can help seek novel diagnostic and prognostic biomarkers for STAD.

In this study, based on several public datasets, the prognostic genes related to *HP* infection in STAD were identified; then, a riskscore model was constructed and validated. Moreover, the correlation between riskscore and tumor immune microenvironment (TIME) was analyzed by Spearman's method. The expression levels of prognostic genes were assessed by single-cell atlas analysis of *HP*-positive STAD. Additionally, the mRNA expression levels of the prognostic genes were verified in STAD cells, and the migration and invasion abilities of STAD cells were performed. This study breaks the limitations of a single dataset, widely aggregates information, greatly enhances the credibility and universality of the research results, uncovers the possible molecular mechanisms behind *HP* infection, and reserves a large number of crucial gene targets for subsequent exploration. Moreover, it explores the relationship between riskscore and immune infiltration from multiple aspects, providing a reference for the further treatment of STAD.

## 2. Material and Methods

### 2.1. Data Collection and Preprocessing

The expression data and clinical phenotype data of STAD were downloaded from The Cancer Genome Atlas (TCGA) database [27], and the samples without survival time and status were removed, ensuring that all patients have a survival time greater than 0 days. The RNA sequencing (RNA-seq) expression profile was converted to transcripts per million (TPM) form and conducted by log2 conversion. Finally, 353 tumor samples were obtained and divided into a train cohort and a test cohort in a 7:3 ratio.

The chip data of four *HP* infection–related datasets, including GSE6143, GSE5081, GSE27411, and GSE60662, was acquired from the Gene Expression Omnibus (GEO) (https://www.ncbi.nlm.nih.gov/geo/) database. Then, the probe was switched to a gene symbol according to the annotation file, and the samples without clinical follow-up information were eliminated.

The single-cell RNA-seq (scRNA-seq) data of GSE150290 was collected from the GEO (https://www.ncbi.nlm.nih.gov/geo/query/acc.cgi?acc=GSE150290) database, containing 22 patients (*HP*-negative = 2 and *HP*-positive = 20). Among them, 21 patients were all taken the tumor tissue and distal normal tissue, while one patient was only taken the tumor tissue. The 10× Genomics platform was applied to construct the database, and the Illumina HiSeq 2500 platform was utilized for sequencing analysis [28].

### 2.2. Calculation of the *HP* Infection Score

Firstly, the gastric infection *HP* gene signature, comprising 39 genes, was obtained from the previous literature [29]. Then, the *HP* infection score in The Cancer Genome Atlas Stomach Adenocarcinoma (TCGA-STAD) cohort was calculated by single-sample gene set enrichment analysis (ssGSEA) using the gene set variation analysis (GSVA) R package [30, 31].

### 2.3. Weighted Gene Coexpression Network Analysis (WGCNA)

The critical modules related to *HP* infection score in STAD were identified using WGCNA R package [32]. The optimal soft threshold (*β*) was determined using the pickSoftThreshold function. The hierarchical clustering was performed to obtain coexpression gene modules, with a criterion of minModulus size = 50 for each module. Then, the module–trait relationships between modules and *HP* infection score were analyzed, and the critical modules with higher correlation were selected for subsequent analysis.

### 2.4. Gene Set Enrichment Analysis (GSEA)

The Kyoto Encyclopedia of Genes and Genomes (KEGG) and Gene Ontology (GO) enrichment analysis were conducted on the genes in critical modules by GSEA using clusterProfiler R package [33]. GO enrichment analysis contained three categories, biological process (BP), cellular component (CC), and molecular function (MF). The KEGG pathways and GO terms with adjusted *p* < 0.05 were deemed to have significant enrichment.

### 2.5. Identification of Differentially Expressed Genes (DEGs)

The DEGs between *HP*-positive and *HP*-negative STAD in GSE6143, GSE5081, GSE27411, and GSE60662 datasets were determined using the limma R package [34]. The screening criteria were log2foldchange(FC) > log2 (1.5) and *p* < 0.05. Then, the *HP* infection–related DEGs were identified by intersecting the module genes and DEGs, and UpSetPlot was drawn.

### 2.6. Construction and Validation of the Riskscore Model

Firstly, the prognostic genes of STAD in train cohort were screened from the *HP* infection–related DEGs by univariate Cox regression analysis (*p* < 0.05). The least absolute shrinkage and selection operator (LASSO) regression analysis was employed for reducing the number of genes using glmnet R package [35]. Then, the prognostic genes relevant to *HP* infection were acquired through stepwise regression analysis, and the riskscore for each patient in the train cohort was calculated according to the following formula [36]:

 $$\begin{array}{c} \text{Riskscore}=\sum \beta i*\text{ExPi} \end{array}$$

*β* denotes the coefficient of key genes in the Cox regression model, and *i* indicates the expression value of key genes.

Based on the optimal threshold of riskscore, the STAD patients were split into high- and low-risk groups, and the overall survival (OS) rate was compared between the two groups by the Kaplan–Meier curve. The receiver operating characteristic (ROC) curve was plotted by timeROC R package [37]. Besides, the robustness of the riskscore model was verified in the test cohort and TCGA-STAD cohort.

### 2.7. Correlation Analysis Between Riskscore and TIME

The infiltration level of 10 immune cells was evaluated by the microenvironment cell population-counter (MCP-counter) R package [38]. The correlation between riskscore and 10 immune cells, together with seven immune checkpoints, was analyzed and displayed by a heatmap.

### 2.8. Single-Cell Atlas of *HP*-Positive STAD

For filtering the scRNA-seq data of GSE150290, Seurat objects were created using the CreateSeuratObject function in Seurat R package [39], retaining the cells with genes number of 200–5000 and mitochondrial gene ratio < 10%. Then, the NormalizeData function was utilized for normalization, principal component analysis (PCA) was employed for dimensionality reduction, and the harmony R package was used to remove the batch effects between different samples [40]. Next, uniform manifold approximation and projection (UMAP) was conducted for dimensionality reduction by the RunUMAP function. The FindNeighbors and FindClusters functions were utilized for cell clustering, with the parameters of dims = 1 : 30 and resolution = 0.1. The cell types were annotated according to the marker genes provided by the CellMarker2.0 database (http://bio-bigdata.hrbmu.edu.cn/CellMarker/).

### 2.9. Cell Culture and Transfection

The normal human gastric epithelial cell line (GES-1, CBP60512) and STAD cell line (AGS, CBP60476) were acquired from Nanjing Cobioer Biotechnology Co., Ltd (Nanjing, China). The cell lines were grown in Dulbecco's modified Eagle medium (DMEM) comprising 10% fetal bovine serum (FBS), and incubated at an incubator under 37°C and 5% CO_2_.

Afterwards, the cell transfection was employed for silencing of *CPVL*. Small interfering (si) RNA targeting *CPVL* (si-*CPVL*) and negative control (si-NC) were synthesized by Shanghai Sangon Biotechnology Co., Ltd (Shanghai, China). The target sequence of si-*CPVL* was GGCTGTTTCGCTCCCTATACAGA. Next, AGS cells were transfected using the Lipofectamine 3000 (Invitrogen, United States) based on the instructions of the manufacturer.

### 2.10. Quantitative Real-Time Polymerase Chain Reaction (qRT-PCR)

Total RNA of GES-1 and AGS cells was isolated by the RNA-easy Isolation Reagent (Vazyme, Nanjing, China). Then, using the FastSCRIPT cDNA Synthesis Kit (Tonbo, 31-5300-0100R), the RNA was reversed into cDNA. Subsequently, to assess the mRNA expression levels of prognostic genes in AGS cells, qRT-PCR was performed with SYBR Premix Ex Taq II (Takara, Shanghai, China). The qRT-PCR procedure was as follows: predenaturation at 95°C for 2 min, followed by 40 cycles of 95°C for 20s, annealing at 58°C for 30s, and extension at 72°C for 20 s. The relevant primer sequences are listed in Table S1. *GAPDH* was used as the reference gene, and the relative expression levels of prognostic genes were calculated by 2^−ΔΔCT^ [41].

### 2.11. Wound Healing Assay

The migration ability of AGS cells was evaluated by wound healing experiment [42]. AGS cells were cultured in a 24-well plate (2 × 10^5^). After 24 h, the DMEM medium was removed, and the cells were washed with phosphate buffer. An aseptic pipette tip was utilized to scratch the cells for generating wound. The plate was continually cultivated for 48 h, and then the representative pictures were captured under the upright microscope (BX53M, Olympus, Japan). The wound closure (percent) of AGS cells was calculated.

### 2.12. Transwell Assay

The invasion capability of AGS cells was tested using transwell experiment [43]. In brief, transwell chambers (8 *μ*m) were added with 250 *μ*L serum-free medium in the upper chamber and with 650 *μ*L medium containing 10% FBS in the lower chamber. The AGS cells were placed in the upper chamber for 24 h. Then, the cells penetrating the lower chamber were fixed with 5% paraformaldehyde for 30 min and stained using 0.1% crystal violet for 15 min. The numbers of invaded AGS cells were observed under the same microscope.

### 2.13. Statistical Analysis

All bioinformatic data were analyzed in R language (Version 3.6.0). Since the Wilcoxon rank-sum test is applicable to continuous variable data, where the distribution does not follow a normal distribution and the samples are independent of each other, this method was chosen to analyze the association between *HP* infection and gene expression. On the other hand, the Spearman correlation analysis was used to evaluate the correlation between the riskscore and the infiltration levels of immune cells in the TIME. This method is suitable for analyzing continuous data with nonlinear and monotonic relationships. Data were presented as mean ± standard deviation, and statistical analysis was conducted by SPSS 17.0. Student's *t*-test was utilized to compare the differences between the two groups. In addition, to reduce the contingency of the experiments, both the qRT-PCR, wound healing, and transwell assays for each group of samples in this study were set with three technical replicates *p* < 0.05 was regarded as statistically significant.

## 3. Results

### 3.1. The Key Modules Related to *HP* Infection in STAD Were Identified by WGCNA

**Figure 1:**
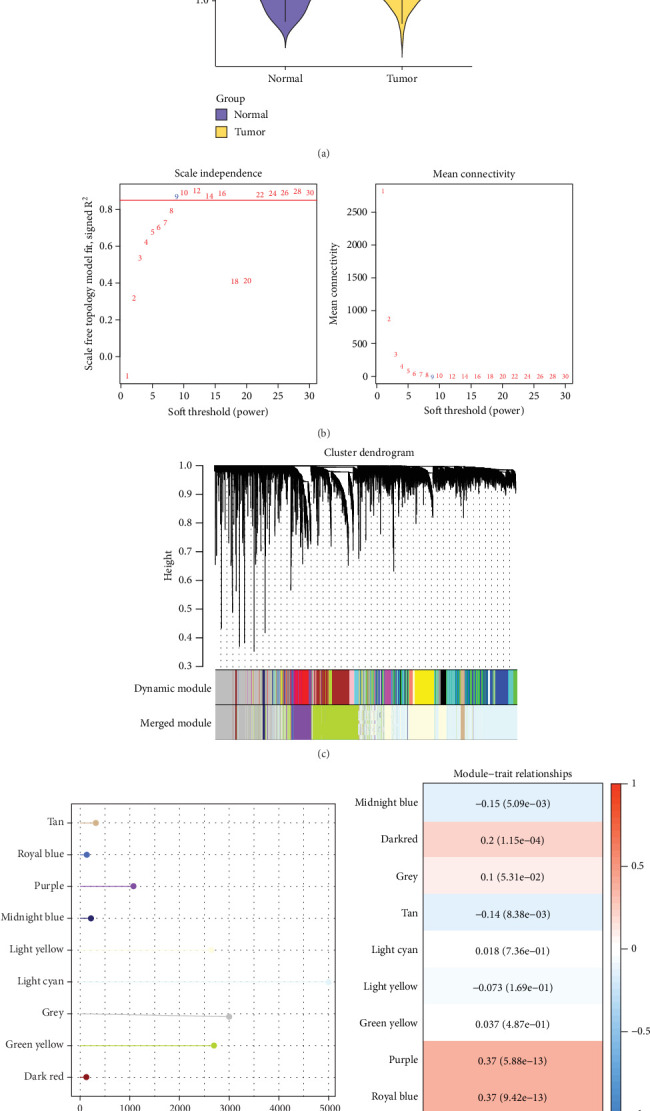
Identification of key modules related to *HP* infection in STAD by WGCNA. (a) *HP* infection score in tumor and normal groups. (b) Screening of soft threshold to construct a topology model. (c) Cluster dendrogram of dynamic module and merged module. (d) Number of genes in each module. (e) Correlation heatmap between modules and *HP* infection score.

In the TCGA-STAD cohort, the *HP* infection score in the tumor group was notably higher than that in the normal group (Figure 1(a)). Then, the *HP* infection score was utilized as a trait for WGCNA. The optimal soft threshold (*β*) was determined to be 9 to construct the topology model (Figure 1(b)). By hierarchical clustering, nine coexpressed gene modules were generated (Figure 1(c)), among which the grey module could not be clustered to other modules. The number of genes in each module was displayed by a lollipop plot (Figure 1(d)). The purple and royal blue modules showed a strong correlation with the *HP* infection score (cor = 0.37) (Figure 1(e)).

### 3.2. Functional Enrichment Analysis Was Performed on the Module Genes

**Figure 2:**
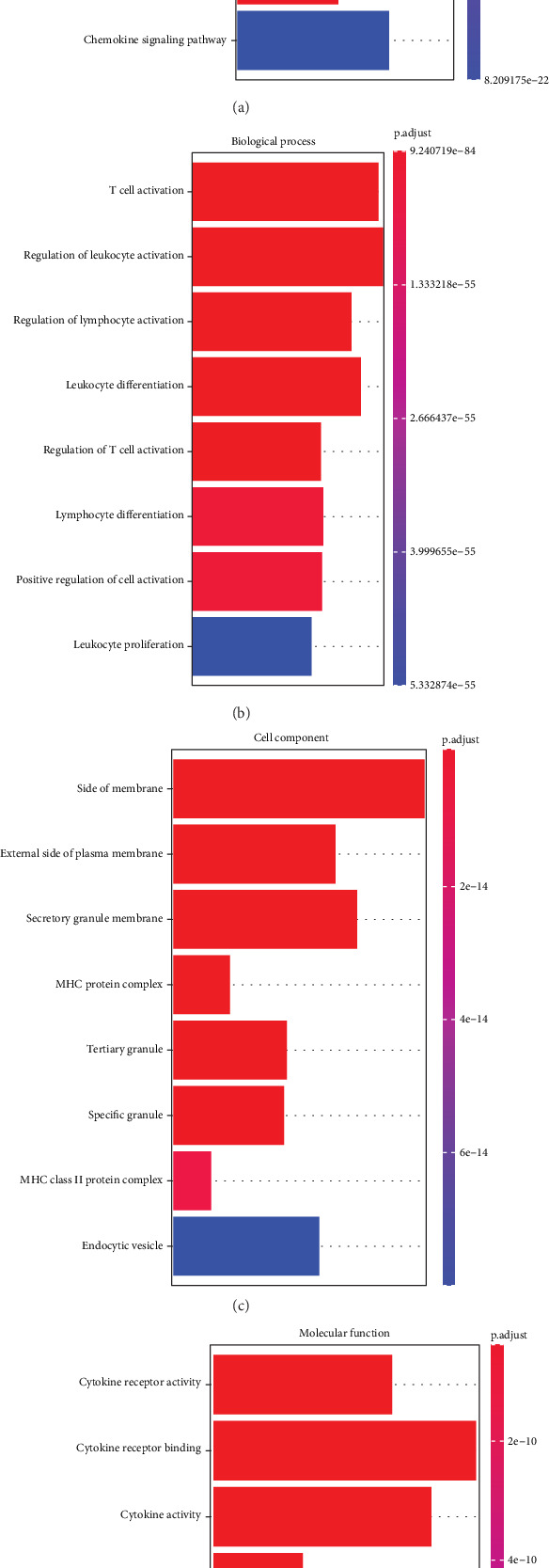
Enrichment analysis of genes in purple and royal blue modules. (a) KEGG pathway enrichment results of module genes. (b) GO functional enrichment results of module genes in biological process (BP). (c) GO functional enrichment results of module genes in cell component (CC). (d) GO functional enrichment results of module genes in molecular function (MF).

For a deep understanding of the biological functions of the purple and royal blue module genes, GO and KEGG enrichment analyses were conducted by GSEA. KEGG analysis revealed that the module genes were significantly enriched in cytokine–cytokine receptor interaction, hematopoietic cell lineage, antigen processing and presentation, chemokine signaling pathway, etc. (Figure 2(a)). GO analysis suggested that in the BP category, the module genes were mainly enriched in multiple immune-related terms, such as T cell activation, regulation of leukocyte activation, regulation of lymphocyte activation, and positive regulation of cell activation (Figure 2(b)). In the CC category, the side of the membrane, secretory granule membrane, MHC protein complex, MHC Class II protein complex, and other terms were markedly enriched (Figure 2(c)). In the MF category, the enriched functions comprised cytokine receptor activity, cytokine receptor binding, MHC protein complex binding, immunoglobulin binding, etc. (Figure 2(d)). These results indicated that the module genes associated with *HP* infection may impact the immune system, thereby influencing the occurrence and development of STAD.

### 3.3. Riskscore Was Established and Exhibited Good Robustness

**Figure 3:**
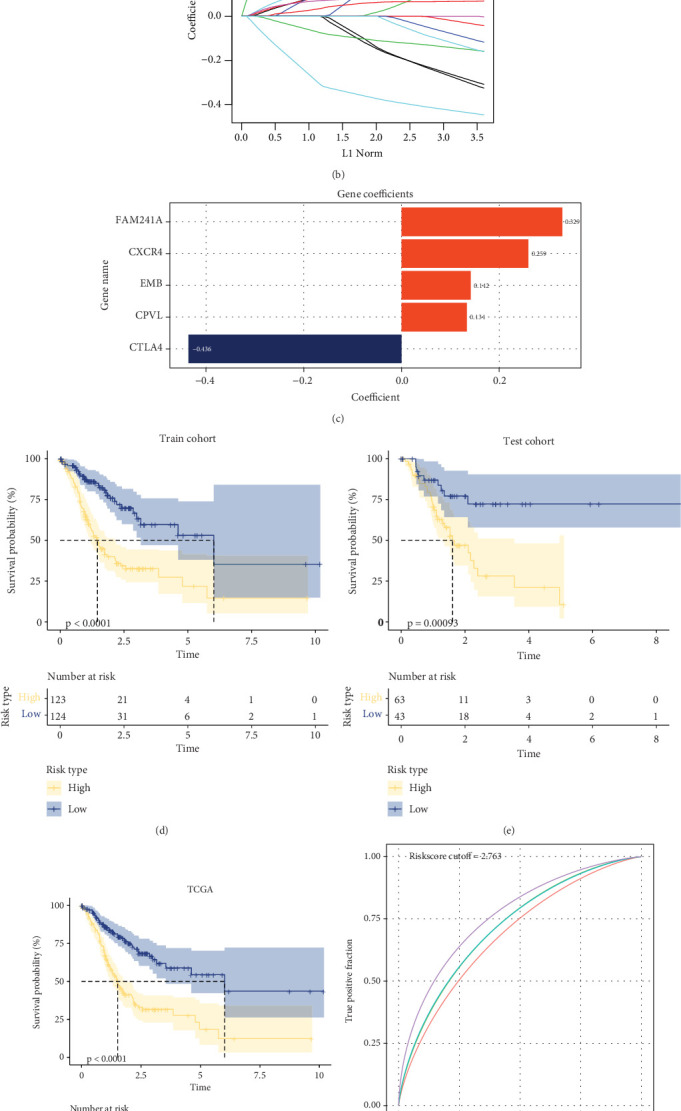
Establishment and validation of the riskscore model. (a) UpSet plot of the intersection between DEGs of four GEO datasets and module genes. (b) LASSO Cox regression analysis to narrow gene number. (c) Coefficient of prognostic genes. (d–f) Kaplan–Meier curves of the riskscore model in the train cohort, test cohort, and TCGA-STAD cohort. (g–i) ROC curves of the riskscore model in the train cohort, test cohort, and TCGA-STAD cohort.

Firstly, the DEGs associated with *HP* infection were obtained by intersecting the module genes and DEGs of GSE6143, GSE5081, GSE27411, and GSE60662, displayed by an UpSet plot (Figure 3(a)). Then, in the train cohort, univariate Cox regression analysis was conducted on the *HP* infection–related DEGs, and LASSO Cox and stepwise regression analysis were further applied to narrow the gene number (Figure 3(b)). Five prognostic genes, including 1 “protective” gene (cytotoxic T lymphocyte-associated antigen 4 (*CTLA4*)) and 4 “risk” genes (*CPVL*, *EMB*, C-X-C chemokine receptor type 4 (*CXCR4*), and *FAM241A*), were screened to construct a riskscore model of “Riskscore = (0.142∗EMB) + (0.134∗CPVL) + (−0.436∗CTLA4) + (0.329∗FAM241A) + (0.259∗CXCR4)” (Figure 3(c)). According to the riskscore, STAD patients were separated into high- and low-risk groups. In the Kaplan–Meier curves, compared with low-risk patients, high-risk patients demonstrated a lower OS rate in the train cohort (Figure 3(d)). Similar trends were also seen in the test cohort and the TCGA-STAD cohort (Figures 3(e) and 3(f)). These findings suggest that STAD patients categorized as high-risk have a poor prognosis. Furthermore, in order to assess the diagnostic accuracy of the riskscore model, the area under the ROC curve (AUC) was calculated. It was observed that the riskscore model was dependable with a 1-year AUC of 0.69, 2-year AUC of 0.71, 3-year AUC of 0.69, and 4-year AUC of 0.75 in the train cohort (Figure 3(g)). Additionally, the riskscore model showed good robustness, which was validated in the test cohort and TCGA-STAD cohort (Figures 3(h) and 3(i)).

### 3.4. The Riskscore Showed Strong Correlation With TIME

**Figure 4:**
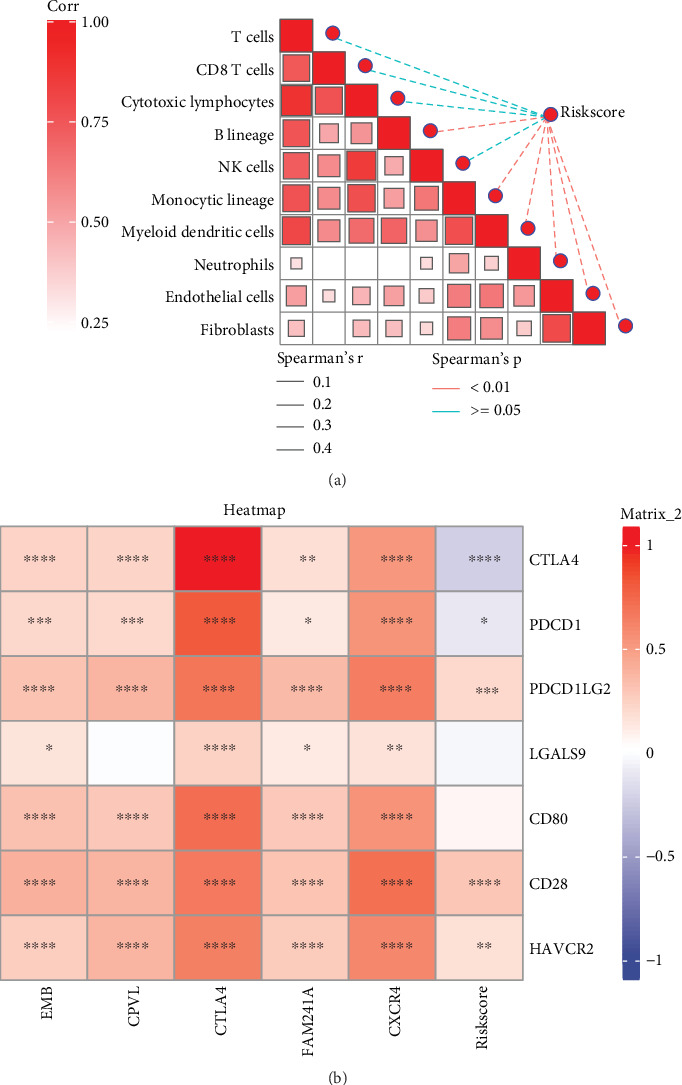
Relationship between riskscore and TIME. (a) Correlation of riskscore and 10 immune cell infiltration by MCP-counter. In this graph, solid lines and dashed lines represent positive correlations and negative correlations, respectively. Red indicates significant correlations (*p* < 0.05), while blue indicates nonsignificant correlations (*p* > 0.05). (b) Correlation of riskscore and seven immune checkpoint genes. ⁣^∗∗∗∗^*p* < 0.0001; ⁣^∗∗∗^*p* < 0.001; ⁣^∗∗^*p* < 0.01; ⁣^∗^*p* < 0.05.

MCP-counter analysis suggested that riskscore exhibited remarkably negative correlation with the infiltration levels of most immune cells, such as B lineage, myeloid dendritic cells, neutrophils, endothelial cells, and fibroblasts (Figure 4(a), *p* < 0.01), manifesting that STAD patients with higher riskscore may have stronger immunosuppressive effect. Besides, riskscore showed a notably positive correlation with several immune checkpoint genes of *CD28*, *PDCD1LG2*, and *HAVCR2* (Figure 4(b)), demonstrating that STAD patients with higher riskscore may be more likely to experience immune escape.

### 3.5. Single-Cell Atlas of *HP*-Positive STAD Was Analyzed

**Figure 5:**
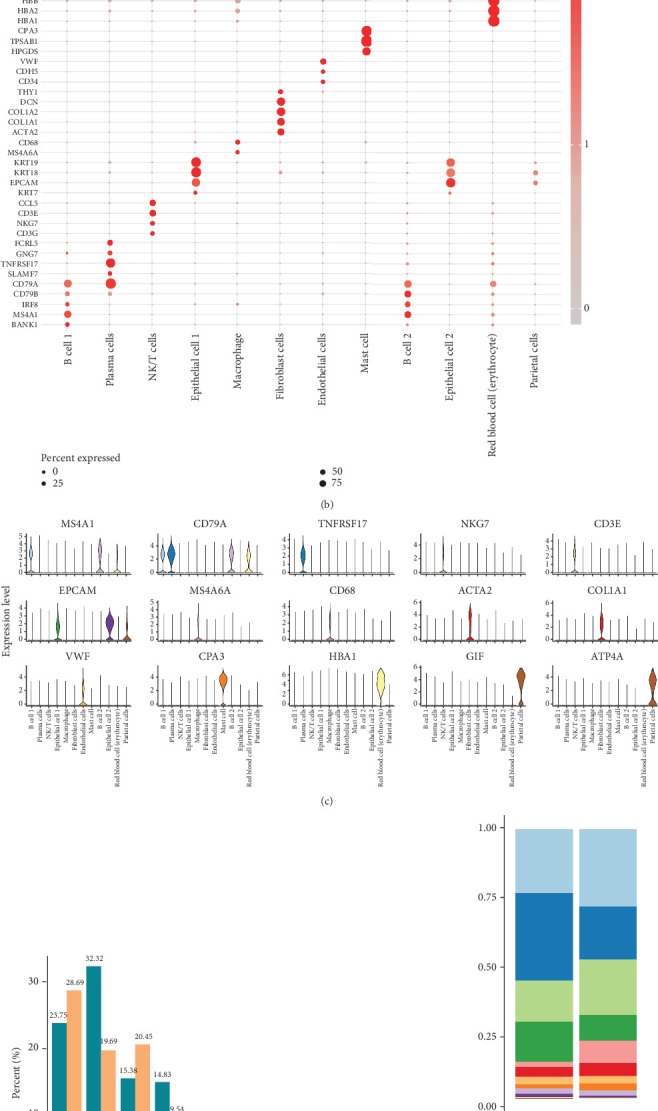
Single-cell atlas of *HP*-positive STAD in GSE150290. (a) UMAP plot of different cell types. (b, c) The expression levels of marker genes in each cell type. (d) The percent of different cell types in the tumor group and adjacent normal group. (e) Stacking plot of each cell type in the tumor group and adjacent normal group.

After cell clustering and annotation of scRNA-seq data in GSE150290, a total of 12 cell types were acquired (Figure 5(a)), including B cell 1 (marked with *MS4A1* and *CD79A*), plasma cells (marked with *CD79A* and *TNFRSF17*), NK/T cells (marked with *NKG7* and *CD3E*), Epithelial Cell 1 (marked with *EPCAM*), macrophage (marked with *MS4A6A* and *CD68*), fibroblast cells (marked with *ACTA2* and *COL1A1*), endothelial cells (marked with *VWF*), mast cell (marked with *CPA3*), B Cell 2 (marked with *MS4A1* and *CD79A*), Endothelial Cell 2 (marked with *EPCAM*), Red blood cell (erythrocyte, marked with *MS4A1*, *CD79A*, and *HBA1*), and parietal cells (marked with *EPCAM*, *GIF*, and *ATP4A*) (Figures 5(b) and 5(c)). Moreover, the percent of B Cell 1, NK/T cells, and macrophage in *HP*-positive STAD group (28.69%, 20.45%, and 8.25%) was higher than that in adjacent normal group (23.75%, 15.38%, and 1.82%), while the percent of plasma cells and Epithelial Cell 1 in tumor group (19.69% and 9.54%) was lower than that in adjacent normal group (32.32% and 14.83%) (Figures 5(d) and 5(e)).

### 3.6. The Expression Level of Prognostic Genes in a Single Cell Was Compared

**Figure 6:**
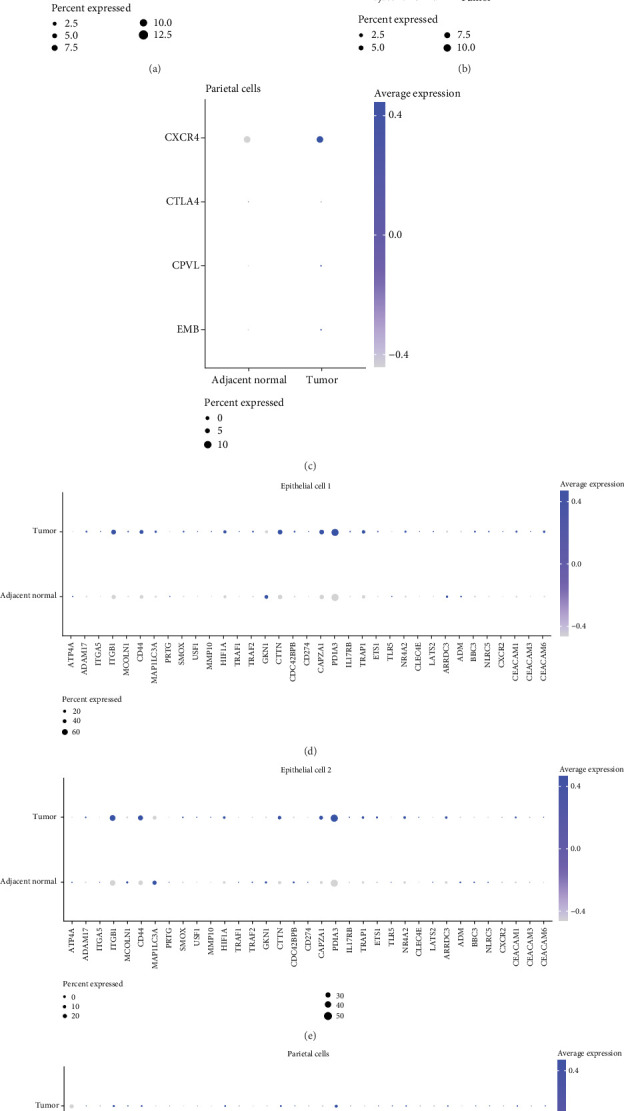
The expression levels of prognostic genes in (a) Epithelial Cell 1, (b) Epithelial Cell 2, and (c) parietal cells. The expression levels of *HP* infection–related genes in (d) Epithelial Cell 1, (e) Epithelial Cell 2, and (f) parietal cells.

*CXCR4* showed high expression level in Epithelial Cell 1, Epithelial Cell 2, and parietal cells of the tumor group (Figures 6(a), 6(b), and 6(c)), revealing that *CXCR4* may play a crucial role in the development of *HP*-positive STAD. In addition, the bubble plot of the expression level of *HP* infection–related genes in single-cell showed that *ITGB1*, *CD44*, *CTTN*, *CAPZA1*, and *PDIA3* were all highly expressed in Epithelial Cell 1 and Epithelial Cell 2 of *HP*-positive STAD (Figures 6(d) and 6(e)). *ATP4A* was highly expressed in the adjacent normal group yet not expressed in the tumor group (Figure 6(f)).

### 3.7. The Silencing of *CPVL* Restrained the Migration and Invasion Abilities of STAD Cells

**Figure 7:**
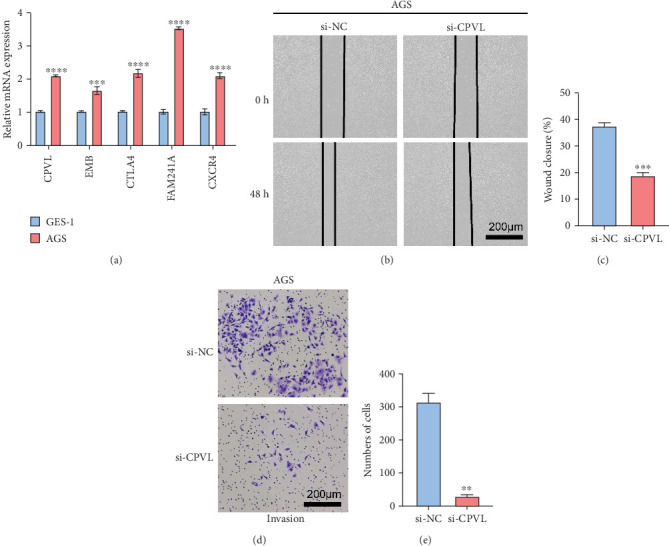
The in vitro validation experiments using STAD cells. (a) Relative mRNA expression levels of five prognostic genes, *CPVL*, *EMB*, *CTLA4*, *FAM241A*, and *CXCR4*, in the STAD cell line (AGS) and normal gastric epithelial cell line (GES-1) via qRT-PCR. (b, c) Wound healing assay to assess the effect of *CPVL* silencing on the migration of STAD cells. (d, e) Transwell assay to evaluate the impact of *CPVL* silencing on the invasion of STAD cells. ⁣^∗∗∗∗^*p* < 0.0001; ⁣^∗∗∗^*p* < 0.001; ⁣^∗∗^*p* < 0.01.

The qRT-PCR validation showed that *CPVL*, *EMB*, *CTLA4*, *FAM241A*, and *CXCR4* were all significantly upregulated expression in AGS cells compared to GES-1 cells (Figure 7(a)). Further, the impact of *CPVL* silencing on the migration and invasion of STAD cells was tested by the wound healing and transwell assays. The silencing of *CPVL* could notably reduce the numbers of migrated and invaded AGS cells (Figures 7(b), 7(c), 7(d), and 7(e)).

## 4. Discussion

STAD is a lethal malignancy with poor therapeutic response and survival probability [44]. *HP* infection was known to be tightly involved in the onset of STAD [45]. In this present study, we found that the *HP* infection score in tumor samples was higher than in normal samples. Importantly, five prognostic genes (*CTLA4*, *CPVL*, *EMB*, *CXCR4*, and *FAM241A*) related to *HP* infection in STAD were identified and applied to establish a riskscore model. The high-risk group had a lower OS rate and poor outcomes, and the model exhibited good robustness. Besides, riskscore showed strong correlation with TIME in STAD. The single-cell atlas of *HP*-positive STAD revealed that *CXCR4* was highly expressed in Epithelial Cell 1, Epithelial Cell 2, and parietal cells of the tumor group.

A growing number of studies have proved that *HP* infection significantly impacts the occurrence and development of STAD, which could serve as a new prognostic biomarker and therapeutic target for STAD patients [46]. In the study of Wu et al. [47], based on 73 *HP* infection–related genes, two different mutation patterns of STAD were identified, with distinct immune infiltration levels and 5-year survival rates, as well as the risk signature and nomogram were established, and low-risk patients showed notable treatment advantages and clinical benefits. Another research by Zhou et al. [48], by microbiome and transcriptome analysis, identified nine differential microbial genera that could classify STAD patients into three subtypes; then, five feature genes (*NTN5*, *MPV17L*, *MPLKIP*, *SIGLEC5*, and *SPAG16*) were screened to construct a prognostic model for predicting the OS rate of STAD patients. In addition, according to Yan et al. [49], a ferroptosis-related gene in *HP* infection, *SOCS1*, could utilized as a candidate prognostic biomarker in STAD, and higher expression of *SOCS1* suggested a bad prognosis in STAD patients. In our study, we found that the *HP* infection score in the tumor group was notably higher than that in the normal group and screened five key prognostic genes (*CTLA4*, *CPVL*, *EMB*, *CXCR4*, and *FAM241A*) from the *HP* infection–related DEGs in STAD to establish a riskscore model. STAD patients were split into high- and low-risk groups, and high-risk patients exhibited lower OS rates and poor prognosis. The model was validated and showed favorable robustness, indicating that this study could provide a new and reliable prognostic model based on *HP* infection–related genes for STAD.

The five key prognostic genes associated with *HP* infection in STAD comprised 1 “protective” gene (*CTLA4*) and 4 “risk” genes (*CPVL*, *EMB*, *FAM241A*, and *CXCR4*), and the riskscore exhibited a negative correlation with the infiltration levels of most immune cells and positive correlation with several immune checkpoint genes. *CTLA4* is an essential immunosuppressive molecule expressed on the T cells together with CD28 [50]. *CTLA4* insufficiency is relevant to the development of GC [51]. *CTLA*4 + 49A/G (rs231775) single nucleotide polymorphism was reported to be a candidate susceptibility marker for GC [52]. Carboxypeptidase vitellogenic like (*CPVL*) is initially discovered in macrophages, involving in the inflammatory protease cascade and peptide pruning of antigen presentation [53]. *CPVL* has been considered as an oncogene that promotes cancer progression, such as glioma [54] and breast cancer [55]. In GC, *CPVL* was found to be a potential prognostic marker [56]. Embigin (*EMB*) is a highly glycosylated protein in the immunoglobulin superfamily [57]. *EMB* was thought to be involved in the progression of GC and correlated with bad prognosis of patients [58]. ANXA2P1/miR-20b-5p/*FAM241A* (C4orf32) was discovered as a tumor suppressive regulatory axe in lung cancer [59]. While there have been no reports on the study of *FAM241A* in GC, *CXCR4* is the most widely expressed chemokine receptor in multiple cancers, including GC, breast cancer, and colorectal cancer [60]. It was reported that the upregulated expression of *CXCR4* was associated with the progression and poor prognosis of STAD [61]. In our study, *CXCR4* was highly expressed in Epithelial Cell 1, Epithelial Cell 2, and parietal cells of the tumor group by the single-cell atlas analysis in *HP*-positive STAD. Simultaneously, the mRNA expression levels of *CPVL*, *EMB*, *CTLA4*, *FAM241A*, and *CXCR4* were markedly increased in AGS cells, and the silencing of *CPVL* could notably suppress migration and invasion abilities of AGS cells. Therefore, we speculate that the five key prognostic genes may be involved in the development of STAD, and *HP* infection–related genes could serve as candidate biomarkers and therapeutic targets for STAD.

The results of immune infiltration analysis showed that riskscore was significantly negatively correlated with the infiltration levels of most immune cells, especially myeloid dendritic cells, neutrophils, endothelial cells, and fibroblasts. Myeloid dendritic cells can uptake, process tumor-associated antigens, and present these antigens to T cells, thus activating the immune response of T cells [62]. In GC, neutrophils may undergo phenotypic changes, for example, transforming from the antitumor N1 to the protumor N2, which contributes to tumor progression and immune suppression [63, 64]. Endothelial cells and fibroblasts are closely related to angiogenesis in GC [65, 66]. These evidences confirm that the infiltration of immune cells may affect the prognosis of STAD. Additionally, the results showed that there was a significant positive correlation between riskscore and key immune checkpoint genes such as CD28, PDCD1LG2, and HAVCR2. Immune checkpoints are a regulatory mechanism in the immune system, which prevent the immune system from being overactivated and causing damage to self-tissues [67]. Among them, the interaction between CD28 and B7 regulates the activity of T cells [68]. Some viruses may encode proteins with a similar structure to B7 molecules to competitively bind to CD28 and block the activation of T cells [69]. PDCD1LG2 binds to programmed cell death protein-1 (PD-1) on T cells to inhibit the immune response, and high expression of PDCD1LG2 will suppress the functions of immune cells [70]. HAVCR2, as an inhibitory receptor, participates in regulating the activity of T cells and can inhibit the proliferation, activation, and cytokine production of T cells by transmitting inhibitory signals [71]. High riskscore is correlated with the high expression of these immune checkpoint genes, which may indicate the activation of tumor immune escape mechanisms. Tumor cells can take advantage of the high expression of immune checkpoints to evade the attack of the immune system. By analyzing the relationship among patients' riskscore, immune infiltration, and immune checkpoints, it is conducive to pinpointing the targets for immunotherapy and devising more precise and personalized immunotherapy regimens for patients, thereby enhancing the treatment efficacy and prolonging patients' survival time. Nevertheless, further in-depth analysis of its specific details is warranted.

There are some limitations in the current study. For example, the sample size of STAD patients in TCGA and GEO databases used in this study was limited. Moreover, the five key prognostic genes were screened only based on public databases, lacking the support of clinical data. Furthermore, the relationship between *HP*-positive STAD and the *CXCR4* gene requires further research and exploration. The specific relationship between tumor cells and immune infiltration, as well as immune checkpoints in STAD, also requires further investigation. In the future, numerous experiments are needed to deeply explore the action mechanisms of *HP* infection–related genes in STAD to verify the results of this study.

## 5. Conclusion

In summary, five prognostic genes associated with *HP* infection in STAD were identified, including 1 “protective” gene (*CTLA4*) and 4 “risk” genes (*CPVL*, *EMB*, *CXCR4*, and *FAM241A*). The riskscore model was constructed and validated, with good robustness in predicting the prognosis of STAD patients. Moreover, riskscore exhibited a strong correlation with TIME. *CXCR4* is highly expressed in Epithelial Cell 1, Epithelial Cell 2, and parietal cells of *HP*-positive STAD. This study could provide an important reference for further exploration of molecular mechanism and potential therapeutic targets of STAD.

## Data Availability

The datasets generated and/or analyzed during the current study are available in the GSE6143 repository (https://www.ncbi.nlm.nih.gov/geo/query/acc.cgi?acc=GSE6143), GSE5081 repository (https://www.ncbi.nlm.nih.gov/geo/query/acc.cgi?acc=GSE5081), GSE27411 repository (https://www.ncbi.nlm.nih.gov/geo/query/acc.cgi?acc=GSE27411), GSE60662 repository (https://www.ncbi.nlm.nih.gov/geo/query/acc.cgi?acc=GSE60662), and GSE150290 repository (https://www.ncbi.nlm.nih.gov/geo/query/acc.cgi?acc=GSE150290).

## Nomenclature

AUC: area under the ROC curve
BP: biological process
CC: cellular component
CPVL: carboxypeptidase vitellogenic like
CTLA4: cytotoxic T lymphocyte–associated antigen 4
CXCR4: C-X-C chemokine receptor type 4
DEGs: differentially expressed genes
EMB: embigin
FC: fold change
GC: gastric cancer
GEO: Gene Expression Omnibus
GO: Gene Ontology
GSEA: gene set enrichment analysis
*HP*: *Helicobacter pylori*
KEGG: Kyoto Encyclopedia of Genes and Genomes
LASSO: least absolute shrinkage and selection operator
MCP-counter: microenvironment cell population-counter
MF: molecular function
OS: overall survival
PCA: principal component analysis
RNA-seq: RNA sequencing
ROC: receiver operating characteristic
scRNA-seq: single-cell RNA-seq
ssGSEA: single-sample gene set enrichment analysis
STAD: stomach adenocarcinoma
TCGA: The Cancer Genome Atlas
TIME: tumor immune microenvironment
TPM: transcripts per million
UMAP: uniform manifold approximation and projection
WGCNA: weighted gene coexpression network analysis
WHO: World Health Organization
DMEM: Dulbecco's modified Eagle medium
FBS: fetal bovine serum
si: small interfering
