# Supplementary material for: A Prognostic Riskscore Model Related to Helicobacter pylori Infection in Stomach Adenocarcinoma
Source: Int J Genomics. 2025 Jan 21;2025:5554610. doi: 10.1155/ijog/5554610 (PMC11779996; doi:10.1155/ijog/5554610)
Supplement: Supporting Information — Additional supporting information can be found online in the Supporting Information section. Table S1: Primer sequences used in qRT-PCR in this study. [file 5554610.f1.docx]

**Supplementary Table 1. Primer sequences used in qRT‑PCR in this study**

| Gene | Primers (5’-3’) |
| --- | --- |
| *CPVL* | Forward: GAATGCATAGAACACATCAG  Reverse: AATTACCTGATGGAAGTCAC |
| *EMB* | Forward: CTTTTACAAGTCCACCTCTC  Reverse: ACTAAAAGAATCACCTCAGC |
| *CTLA4* | Forward: AGATTCTGACTTCCTCCTCT  Reverse: ACATAGACCCCTGTTGTAAG |
| *FAM241A* | Forward: CGGCAGGGACCGAGTGGGAT  Reverse: GCTCACCAGTGTGGTTCTGTGA |
| *CXCR4* | Forward: CTCCTCTTTGTCATCACGCTTCC |
|  | Reverse: GGATGAGGACACTGCTGTAGAG |
| *GAPDH* | Forward: ATTGACCTCAACTACATGGT  Reverse: CATACTTCTCATGGTTCACA |
